# Shared and Unique Disease Pathways in Amyotrophic Lateral Sclerosis and Parkinson’s Disease Unveiled in Peripheral Blood Mononuclear Cells

**DOI:** 10.1021/acschemneuro.3c00629

**Published:** 2023-11-08

**Authors:** Marta Lualdi, Federico Casale, Mario Giorgio Rizzone, Maurizio Zibetti, Chiara Monti, Ilaria Colugnat, Andrea Calvo, Giovanni De Marco, Cristina Moglia, Giuseppe Fuda, Cristoforo Comi, Adriano Chiò, Leonardo Lopiano, Mauro Fasano, Tiziana Alberio

**Affiliations:** † Department of Science and High Technology and Center for Research in Neuroscience, University of Insubria, I-21052 Busto Arsizio, Varese, Italy; ‡ Neurology 1, ALS Expert Center, “Rita Levi Montalcini” Department of Neuroscience, University of Torino, and AOU Città della Salute e della Scienza, I-10126 Torino, Italy; § “Rita Levi Montalcini” Department of Neuroscience, University of Torino, and AOU Città della Salute e della Scienza, I-10126 Torino, Italy; ∥ Department of Translational Medicine, University of Piemonte Orientale, and Sant’Andrea Hospital, I-13100 Vercelli, Italy

**Keywords:** amyotrophic lateral sclerosis, Parkinson’s disease, peripheral blood mononuclear cells, two-dimensional electrophoresis, sPLS-DA, fibrinogen

## Abstract

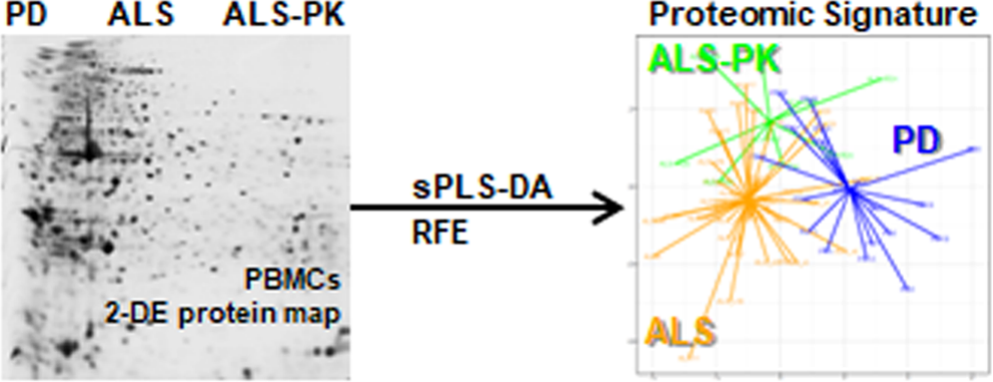

Recent evidence supports an association between amyotrophic lateral sclerosis (ALS) and Parkinson’s disease (PD). Indeed, prospective population-based studies demonstrated that about one-third of ALS patients develop parkinsonian (PK) signs, even though different neuronal circuitries are involved. In this context, proteomics represents a valuable tool to identify unique and shared pathological pathways. Here, we used two-dimensional electrophoresis to obtain the proteomic profile of peripheral blood mononuclear cells (PBMCs) from PD and ALS patients including a small cohort of ALS patients with parkinsonian signs (ALS-PK). After the removal of protein spots correlating with confounding factors, we applied a sparse partial least square discriminant analysis followed by recursive feature elimination to obtain two protein classifiers able to discriminate (i) PD and ALS patients (30 spots) and (ii) ALS-PK patients among all ALS subjects (20 spots). Functionally, the glycolysis pathway was significantly overrepresented in the first signature, while extracellular interactions and intracellular signaling were enriched in the second signature. These results represent molecular evidence at the periphery for the classification of ALS-PK as ALS patients that manifest parkinsonian signs, rather than comorbid patients suffering from both ALS and PD. Moreover, we confirmed that low levels of fibrinogen in PBMCs is a characteristic feature of PD, also when compared with another movement disorder. Collectively, we provide evidence that peripheral protein signatures are a tool to differentially investigate neurodegenerative diseases and highlight altered biochemical pathways.

## 1 Introduction

Amyotrophic lateral sclerosis (ALS) is a neurodegenerative disease (ND) characterized by the progressive loss of motor neurons in the brain and spinal cord.^1−3^ Motor neuron loss causes inability to move, eat, and speak, eventually leading to paralysis and death by respiratory failure within 3–10 years from disease onset. Approximately half of the ALS patients also develop cognitive impairment, ranging from mild difficulties with thinking to severe frontotemporal dementia (FTD).^4,5^ Thus, ALS represents a complex disease, encompassing a spectrum of different clinical phenotypes.^6^

Roughly 50 years ago the Brait–Fahn–Schwartz disease was described for the first time as a disorder characterized by the coexistence of ALS and Parkinson’s disease (PD).^7^ PD is the second most frequent ND worldwide and it is characterized by the loss of nigral dopaminergic neurons, leading to the onset of hallmark motor symptoms, i.e., resting tremor, bradykinesia, and rigidity.^8^ However, nonmotor symptoms are also present in PD patients (e.g., anosmia, cognitive dysfunction, sleep-awake dysregulation, dysautonomia, depression, constipation), related to the functional impairment of different brain circuitries.^9,10^ Convincing evidence was collected supporting an association between ALS and PD. Indeed, cross-sectional studies have demonstrated the presence of parkinsonian (PK) signs in ALS patients, with a frequency ranging from 5 to 17%.^11^ More recently, Calvo and co-workers published the results of a prospective population-based study, involving more than 100 ALS patients consecutively enrolled and prospectively followed over a 2-year period, aimed at investigating the occurrence of parkinsonian features in ALS patients.^12^ As a result, about 30% of the ALS patients showed PK signs (i.e., bradykinesia and rigidity and/or resting tremor). ALS patients with PK signs were more frequently male, but they did not differ for any other demographic, clinical, or neuropsychological factors compared to ALS patients without PK signs. Interestingly, neuroimaging of the brain revealed that PK signs were related to the involvement of pathways other than classical nigrostriatal ones. Indeed, altered neuronal functions emerged in other brain regions related to motor function, i.e., relative hypermetabolism in frontal regions, hypometabolism in cerebellum, reduced cortical thickness in the left precentral region, increased fractional anisotropy in the retrolenticular part of the internal capsule, and reduced fractional anisotropy in the sagittal stratum.^12^ The latter evidence further supports the hypothesis of ALS as a complex multisystem disease.

Due to the complexity of both ALS and PD, the use of untargeted omics approaches (e.g., proteomics and metabolomics) represents an effective strategy to disentangle disease pathobiology and to possibly identify some unique and/or shared features.^13^ Indeed, the identification of disease-specific biomarkers using omics techniques promises to (i) unveil hidden disease mechanisms, (ii) allow for patients’ stratification, and (iii) direct the choice of the proper therapeutic intervention. In the context of NDs, both gel- and mass spectrometry-based proteomics approaches have been successfully applied to this purpose, using autoptic brain specimens, peripheral tissues, and biofluids.^14^ In particular, two-dimensional electrophoresis (2-DE) still represents a powerful approach in proteomics to investigate the presence of different proteoforms belonging to the same protein, which exert different functions and whose levels can specifically change due to the presence of pathological conditions.

Peripheral blood mononuclear cells (PBMCs) are widely recognized as a good source for biomarker discovery studies.^15^ Indeed, compared to cerebrospinal fluid (CSF), which is considered the best source for biomarkers in the field of NDs, PBMCs are inexpensive and easy to collect with no discomfort for the patients. Moreover, compared to plasma and serum, which are also easy to collect, PBMCs offer the possibility to detect and quantify low-abundance proteins, which are usually neglected due to the wide dynamic range of protein concentrations in blood-derived fluids. Eventually, several recent studies revealed that PBMCs (i.e., T and B lymphocytes, natural killer cells, and monocytes) nicely recapitulate the conditions of the surrounding tissues.^16,17^ We used 2-DE to compare the proteome of T-lymphocytes from PD patients with that of control subjects, which led us to the identification of a panel of 17 proteins that performed well as a classifier for PD.^18^ The main limitation of this protein panel was that PD patients were compared with healthy subjects.

In the present study, we used a gel-based proteomics approach (2-DE), to analyze the proteomic profile of PBMCs from ALS patients with or without PK signs compared to PD patients. This study was aimed at (i) highlighting common and unique features in ALS and PD, and (ii) identifying a protein signature related to the occurrence of PK signs in ALS patients. Moreover, we aimed at verifying if some proteins belonging to our 17-proteins classifiers were still able to discriminate PD when compared to patients affected by a different movement disorder (ALS).

## 2 Results and Discussion

### 2.1 Proteomic Profiling of PBMCs by Two-Dimensional Electrophoresis

PD and ALS may show a wide spectrum of clinical phenotypes affecting different neuronal cell types. The identification of specific and general pathways of neurodegeneration,^19^ as well as that of specific and general biomarkers for NDs, is a major need. PBMCs are a compelling source of biomarkers for NDs since they witness at the periphery several biochemical alterations that take place in the brain of both PD^20^ and ALS patients.^21^ In this frame, we coupled here a standard biochemical technique (2-DE of PBMCs) with a systems biology-based multivariate approach to identify specific protein signatures that discriminate two movement disorders (ALS and PD) and a peculiar clinical subtype (i.e., ALS patients with PK signs). This study design also allowed us to overcome the main bias of conventional biomarker discovery studies, where diseased patients are often compared with control subjects, whose heterogeneity is typically higher. Therefore, we verified here whether a peripheral protein signature could characterize PD even when compared with ALS, and vice versa. Moreover, since PK signs frequently occur in ALS patients,^12^ we decided to also include this peculiar ALS clinical subtype in our study (ALS-PK). From a genetic point of view, it has been demonstrated that the mutation of several genes (e.g., *VCP*, *CHMP2B*, *PFN1*, and *C9ORF72*) induces high susceptibility to develop forms of comorbidity between ALS and PD.^22,23^ Even if ALS-PK subjects are actually ALS patients, they may carry mutations in genes previously linked to ALS or PD only, or display a peculiar genetic background.^24^

Based on this rationale, patients were enrolled in four groups: (i) *de novo* PD patients (PD, *n* = 20), (ii) *de novo* ALS patients (ALS_n, *n* = 20), (iii) ALS patients already under riluzole treatment (ALS_r, *n* = 20), and (iv) ALS patients with parkinsonian symptoms (ALS-PK, *n* = 9). We enrolled both ALS_n and ALS_r because ALS-PK subjects were either in treatment or not in treatment with riluzole at the time of withdrawal. On the other hand, since ALS-PK patients were not in treatment with drugs for PD, the enrolled PD patients were not yet under pharmacological treatment. Of note, we have already demonstrated that L-DOPA and DA agonists induce modifications in the proteome of T-lymphocytes.^25^

Protein expression profiles of PBMCs from all patients (*n* = 69) were obtained by 2-DE. A total of about 400 spots were detected on the 2-DE gels. Fifty spots were present in all gels. The sum of the volumes of these common spots was computed in each 2-DE gel and it was plotted to verify a Gaussian distribution (Figure S1). Then, this value was used for normalization after single spots quantification. After the visualization of the distribution of missing values (Figure S2), only those spots that were observed in at least 65% of all gels (i.e., in 45 gels out of 69) were retained for further analysis, namely, 324 spots (Table S1). Moreover, the interindividual variability was evaluated in each group. To this purpose, Pearson linear correlation of spot volumes after logarithmic transformation was computed in pairs of 2-DE gels in the same group (Figure S3). All gel pair comparisons showed linear behavior. Therefore, all gels were retained, and spot volumes were used for the analysis.

All maps (*n* = 69) were also screened to identify proteins whose changes were linked to possible confounding factors, i.e., gender, age, and drug treatment. Seven spots showed significant differences between male and female patients by the Wilcoxon test (*p* < 0.01) (Figure S4), 21 spots showed significant (*p* < 0.01) Pearson linear correlation between spot volume and the age of the patients (Figure S5), and 13 spots showed significant differences between ALS_r and ALS_n by the Student’s *t* test (*p* < 0.01) (Figure S6). The volume of these 13 spots did not correlate with the ALSFRS-R score (Table S2). All spots whose volumes significantly correlated with any confounding factors analyzed (*n* = 39) were then removed.

### 2.2 Supervised Clustering of Patients’ Groups by sPLS-DA

**Figure 1:**
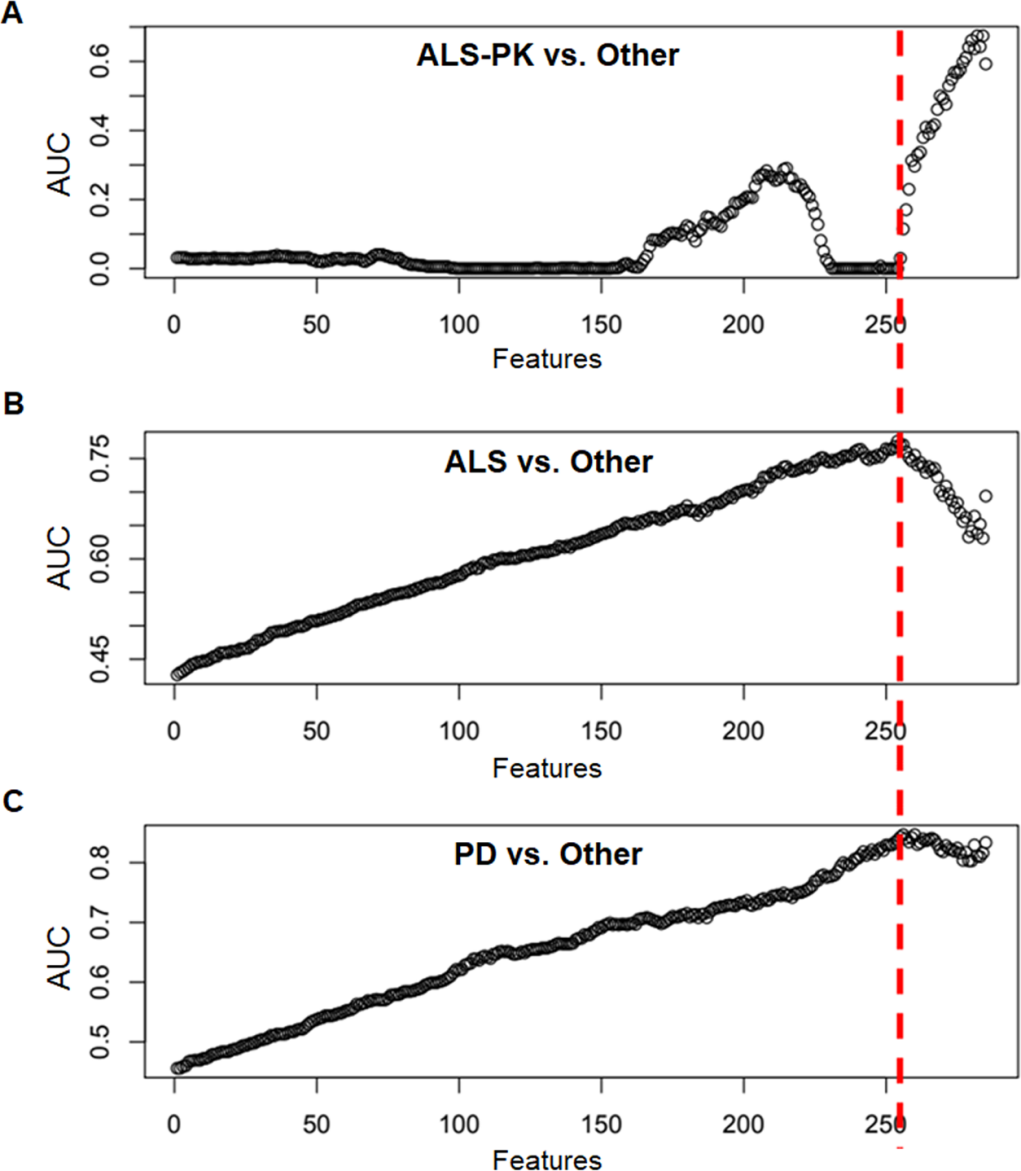
ROC AUC values of sPLS-DA component 1 obtained for each model during recursive feature elimination. (A) ALS-PK patients against the other two groups (ALS plus PD). (B) ALS patients against the other two groups (ALS-PK plus PD). (C) PD patients against the other two groups (ALS plus ALS-PK). The red dotted line represents the threshold of 255 features.

After the elimination of spots associated with confounding factors, the remaining 285 spots were used to perform a standard differential expression analysis (DEP). After multiple testing correction, no significant differences were observed among the three groups for the single proteoforms. Therefore, we decided to employ a supervised multivariate approach aimed at identifying a protein signature that could discriminate the patients’ groups. To this purpose, the 285 spots were used to build a sparse Partial Least Square Discriminant Analysis (sPLS-DA) classifier for ALS, ALS-PK, and PD groups. The model was cross-validated using the leave-one-out procedure, and three receiver operating characteristic (ROC) curves were generated. The model was refined by recursive feature elimination (RFE) so that 284 iterative models were generated in which the spot with the lowest loading was eliminated. The area under the ROC curve was used as a figure of merit to monitor the effectiveness of the RFE procedure. As shown in Figure 1A, no separation was observed for ALS-PK patients vs all other patients, suggesting that a characteristic PBMCs protein signature for ALS-PK could not be identified with this approach. In other words, a combination of features that allowed ALS-PK patients to be separated from all of the other patients was not found. This result is not surprising if we consider that ALS-PK patients are actually clinically classified as ALS. By contrast, a good area under the curve (AUC) value was observed for (i) ALS vs the other two groups (PD plus ALS-PK), and (ii) PD vs the other two groups (ALS plus ALS-PK), with a maximum when 255 features were removed, and 30 spots were retained, according to their loadings in the sPLS-DA (Figure 1B,C). Since the ALS-PK patients do not contribute to the model, the other two ROC curves show the effectiveness of the same separation, i.e., PD vs ALS.

**Figure 2:**
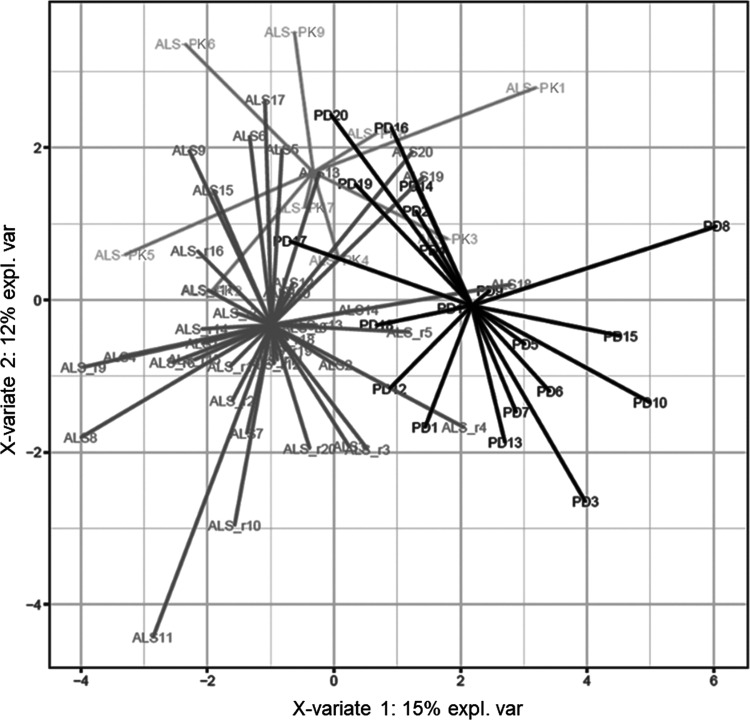
Score plot of the sPLS-DA classification based on the 30-spots signature after leave-one-out cross-validation. Black: PD patients. Shades of gray: ALS and ALS-PK patients.

The residual features set (*n* = 30) generated a discriminant model whose score plot is shown in Figure 2. Remarkably, the first discriminant function separated quite well ALS (including both ALS and ALS-PK subgroups) from PD, whereas ALS-PK patients overlap even if two functions were used. Thus, this molecular signature represents a good discriminant model between PD and ALS patients, unveiling important features that can be characteristic of one movement disorder compared to the other. Moreover, the signature provides further support for the classification of ALS-PK subjects as ALS with additional symptoms rather than patients suffering from both ALS and PD. On these bases, the onset of PK signs in these patients might be due to alterations in molecular pathways and neuronal circuitries other than those involved in PD pathogenesis.

### 2.3 Identification of the Selected Spot Set and Functional Interpretation of the Signature

**Figure 3:**
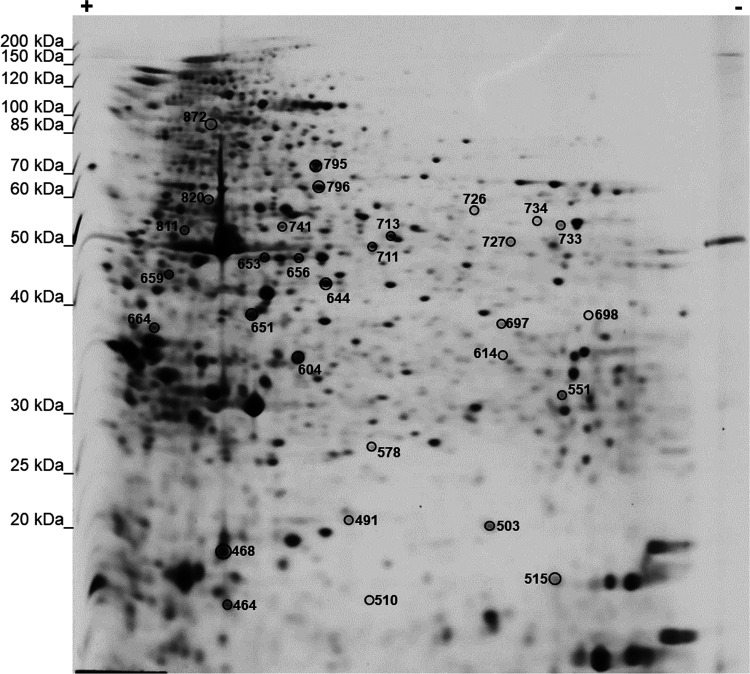
Representative 2-DE map showing the number identifiers of the 30 spots in the signature.

Proteins corresponding to the 30 spots included in the signature (Figure 3) were excised from gels, digested with trypsin, and identified by LC-MS/MS. Table 1 reports protein identifiers for each spot, as labeled in Figure 3.

**Table 1:** Identification of Protein Spots

spot number	gene	UniProt ID	protein	MW (kDa)	isoelectric point	loadings
464	COTL1	Q14019	coactosin-like protein	15.9	5.5	–0.188
468	TAGLN2	P37802	transgelin-2	22.4	8.4	0.183
491	NME1	P15531	nucleoside diphosphate kinase A	17.2	5.8	–0.205
503	HSPA8	P11142	heat shock cognate 71 kDa protein	70.9	5.4	–0.223
510	PFDN5	Q99471	prefoldin subunit 5	17.3	5.9	–0.155
515	PPIA	P62937	peptidyl-prolyl cis–trans isomerase A	18.0	7.7	–0.198
551	PGAM1	P18669	phosphoglycerate mutase 1	28.8	6.7	–0.176
578	ABHD14B	Q96IU4	protein ABHD14B	22.3	5.9	–0.216
604	CAPZB	P47756	F-actin-capping protein subunit β	31.3	5.4	–0.164
614	PNP	P00491	purine nucleoside phosphorylase	32.1	6.5	–0.207
644	TUBB2A	Q13885	tubulin β-2A chain	49.7	4.8	–0.183
651	TUBB	P07437	tubulin β chain	49.7	4.8	–0.151
653	MSN	P26038	moesin	67.8	6.1	–0.196
656	MSN	P26038	moesin	67.8	6.1	–0.162
659	LMNB1	P20700	lamin-B1	66.4	5.1	–0.278
664	TPM1	P09493	tropomyosin α-1 chain	32.7	4.7	0.273
697	ENO1	P06733	α-enolase	47.2	7.0	–0.153
698	MGLL	Q99685	monoglyceride lipase	33.3	6.5	–0.141
711	PKM	P14618	pyruvate kinase PKM	57.9	7.9	0.139
713	FGG	P02679	fibrinogen γ chain	51.5	5.4	–0.166
726	VCL	P18206	vinculin	124	5.5	–0.144
727	FGB	P02675	fibrinogen β chain	56	8.5	–0.222
733	ENO1	P06733	α-enolase	47.2	7.0	–0.152
734	ENO1	P06733	α-enolase	47.2	7.0	–0.153
741	TUBA1A	Q71U36	tubulin α-1A chain	50.1	4.9	–0.162
795	VCL	P18206	vinculin	124	5.5	0.171
796	PDIA3	P30101	protein disulfide-isomerase A3	56.8	5.9	–0.147
811	VIM	P08670	vimentin	53.7	5.1	–0.141
820	VIM	P08670	vimentin	53.7	5.1	–0.169
872	VCL	P18206	vinculin	124	5.5	–0.153

As already mentioned, this 30-spots signature mainly discriminates between PD patients and ALS patients. In this signature, two spots belonged to the fibrinogen complex, fibrinogen protein β and γ chains (FGB, FGG). Since they have already been associated with biomarker signatures related to PD,^18,26,27^ we verified whether they still represented a discriminative feature for PD patients, even when compared with ALS. Both FGB and FGG were significantly underrepresented in PD (two-tailed Welch Student’s *t* test, *p* < 0.05). We have originally demonstrated that high amounts of fibrinogen are present in isolated lymphocytes, particularly in T-cells, and that a lower abundance of fibrinogen in T-cells contributed to a protein biomarker signature for PD.^18,26^ Moreover, we described fibrinogen internalization dynamics within lymphocyte cells and recently reported that FcRn receptor is involved in the rescue of fibrinogen from lysosomal degradation.^28,29^ Therefore, the fibrinogen level in PBMCs remains a discriminant feature of PD and represents a feature that deserves further molecular characterization and that might have diagnostic exploitation.

Of note, among the 30 spots of the signature, some proteins appeared more than once, namely, moesin, α-enolase, vimentin, and vinculin. More in detail, moesin was detected twice (spots #653 and #656), and the two spots were slightly shifted in terms of isoelectric point. Several PTMs that can cause the observed shift are known for this protein: phosphoserine, phosphotyrosine, phosphothreonine, S-nitrosocysteine, N6-acetyllysine, N6-succinyllysine. The phosphorylation of moesin on its C-terminal threonine leads to interaction with F-actin and cytoskeletal rearrangement, which regulates many cellular processes, including cell shape determination, membrane transport, and signal transduction. The role of moesin is important in immunity acting on both T and B lymphocyte homeostasis and self-tolerance.^30,31^ However, we did not observe any significant differences in the amount of the two proteoforms (spot #653 and #656) across patients’ groups, thus suggesting a change in the total amount of the protein rather than a different processing of it in PD patients compared with ALS.

Alpha-enolase was also detected twice (spot #733, #734), with shifts of both MW and isoelectric point. Several PTMs are known also for this protein, mainly phosphorylation and acetylation, at several residues, which play an important role in the reversible regulation of protein function. Phosphorylation seems to be decreased with age, while carbonylation has been detected under oxidative stress. It is still unclear how the PTMs of α-enolase can affect its catalytic activity, subcellular localization, protein stability, and the ability to dimerize or interact with other molecules. Of note, beyond its role as a key glycolytic enzyme, α-enolase is also expressed on the surface of different cell types where it acts as a plasminogen receptor and it is now recognized as a marker of pathological stress in several diseases, including Alzheimer’s disease, cancer, heart failure, and rheumatoid arthritis.^32^ However, our results showed that all proteoforms contributed to group discrimination with negative loadings, suggesting that there was not a specific role associated with single proteoforms.

Vinculin was detected three times (spot #726, #795, and #872) with shifts of both MW and isoelectric point. Vinculin is a F-actin-binding protein involved in both cell–matrix and cell–cell adhesion. It is a huge (1134 amino acids) and highly abundant protein, which could partially explain why we found several proteoforms at different MW and isoelectric point values. Phosphorylation on Tyr-1133 in activated platelets is known to affect head–tail interactions and cell spreading. The protein is also acetylated, mainly by myristic acid but also by palmitic acid.^33^ Also for this protein, however, we did not observe a different contribution on group separation linked to the three different proteoforms.

**Figure 4:**
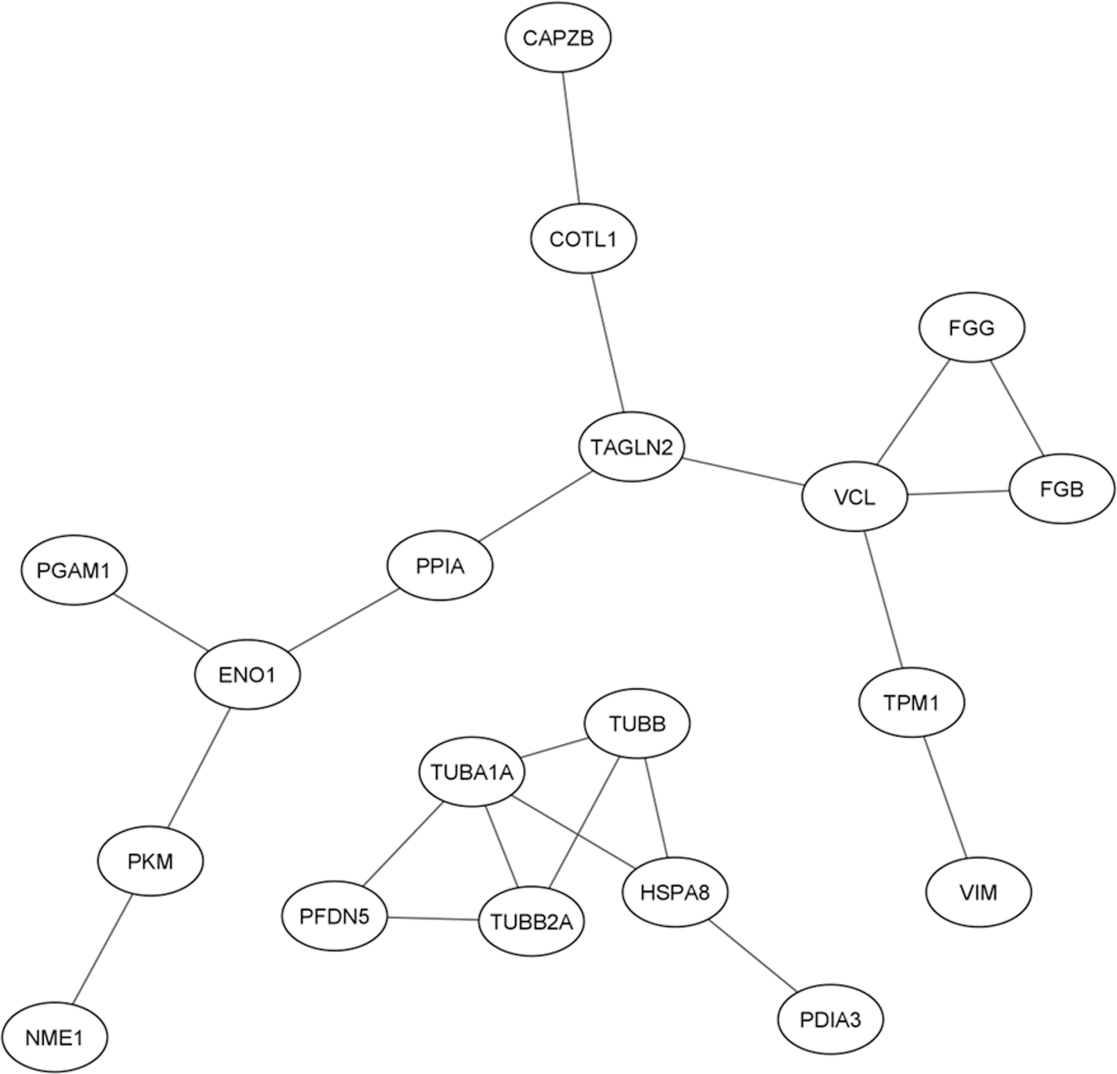
PPI network generated by String db. The network, separated into 2 subclusters, has been generated by using evidence coming from experimental data or retrieved by databases. PPI enrichment *p*-value: 7.31 × 10^–12^.

Vimentin, eventually, was detected twice (spot #811, #820) with a very small shift of both MW and isoelectric point. Vimentins are intermediate filaments found attached to the nucleus, endoplasmic reticulum, and mitochondria in various nonepithelial cells. Phosphorylation is enhanced when the vimentin filaments are significantly reorganized. S-nitrosylation is induced by IFN-γ and oxidatively modified LDL.^34,35^ Again, from our results we did not have any evidence supporting a possible different role of the two proteoforms identified. As a general consideration, the signature should be considered as a whole to classify our patients’ groups. Thus, what is important is the possible contribution of all of these proteins in common molecular pathways. Therefore, to functionally interpret the signature, the 30 IDs have been used to build a protein–protein interaction (PPI) network using the String database. Edges have been added only if experimentally determined or retrieved by databases (Figure 4). The network enrichment *p*-value (7.31 × 10^–12^) means that proteins of the input list have more interactions among themselves than would be expected for a random set of proteins of similar size drawn from the genome. This indicates that the proteins are at least partially biologically connected and participate to the same biological pathways.

To identify these biological pathways, the list of 30 IDs has been analyzed by over-representation analysis (ORA), using Reactome as a reference database. After results’ redundancy reduction by weighted set cover, the enriched significant pathways resulted in “Immune System”, “Hemostasis”, “Prefoldin-mediated transfer of substrate to CCT/TriC”, and “Glycolysis” (Table 2). The enrichment of the glycolytic pathway was due to Phosphoglycerate mutase 1, α-enolase and Pyruvate kinase. The change of the metabolic function of the nervous system and peripheral cells is a common pathophysiological change in all NDs. However, the discrimination between ALS and PD by the sPLS-DA attests that these alterations are different, may involve different molecular factors, and be in opposite directions. Indeed, it has already been reviewed that glycolytic flux may be increased in PD patients’ white cells and decreased in ALS patients.^36^ Therefore, the use of metabolic biomarkers in the diagnosis and monitoring of NDs is a yet underutilized opportunity that deserves further deepening.

**Table 2:** Functional Enrichment Analysis Results: 30 Spot Signature (PD vs ALS)

gene set	description	size	expect	ratio	FDR
R-HSA-68256	immune system	1997	4.54	3.74	0.000073946
R-HSA-09582	hemostasis	620	1.41	6.38	0.0028367
R-HSA-89957	prefoldin-mediated transfer of substrate to CCT/TriC	26	0.059	50.73	0.0056087
R-HSA-70171	glycolysis	70	0.16	18.84	0.035386

### 2.4 Feature Selection to Build a Classifier for ALS Patients with PK Signs

**Figure 5:**
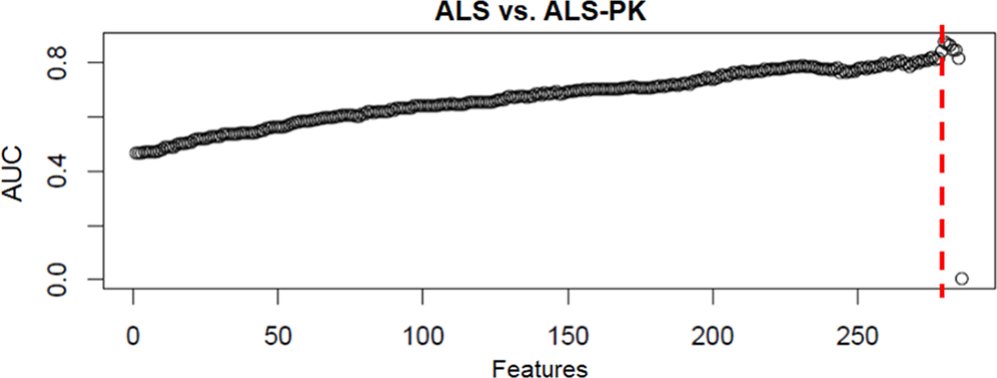
ROC AUC values of sPLS-DA component 1 obtained for each model during recursive feature elimination. The red dotted line represents the threshold for the selection of the six features.

As stated before, the previously generated signature was able to discriminate PD patients from ALS patients, but it was not capable of separating ALS-PK patients from the other two groups. Thus, we decided to use a different feature selection procedure to identify a protein signature related to the presence of parkinsonian signs in ALS patients. To this purpose, we excluded the PD patients’ group, and we only compared ALS-PK patients with the main ALS group. As a first attempt, 285 spots were used to construct an sPLS-DA between ALS and ALS-PK patients. The RFE procedure did not provide good results, with a final selection of just six spots (Figure 5).

This result was not completely unexpected, since ALS-PK actually represents a clinical subgroup of ALS. However, this can also be due to the lower number of ALS-PK patients compared to the ALS group (*n* = 9 and *n* = 40, respectively). Based on a preliminary power analysis, we already computed that the minimum number of patients needed in each group was 20. Unfortunately, we succeeded in enrolling only 9 ALS-PK patients in the project time frame, also due to the lower prevalence of this condition.

**Figure 6:**
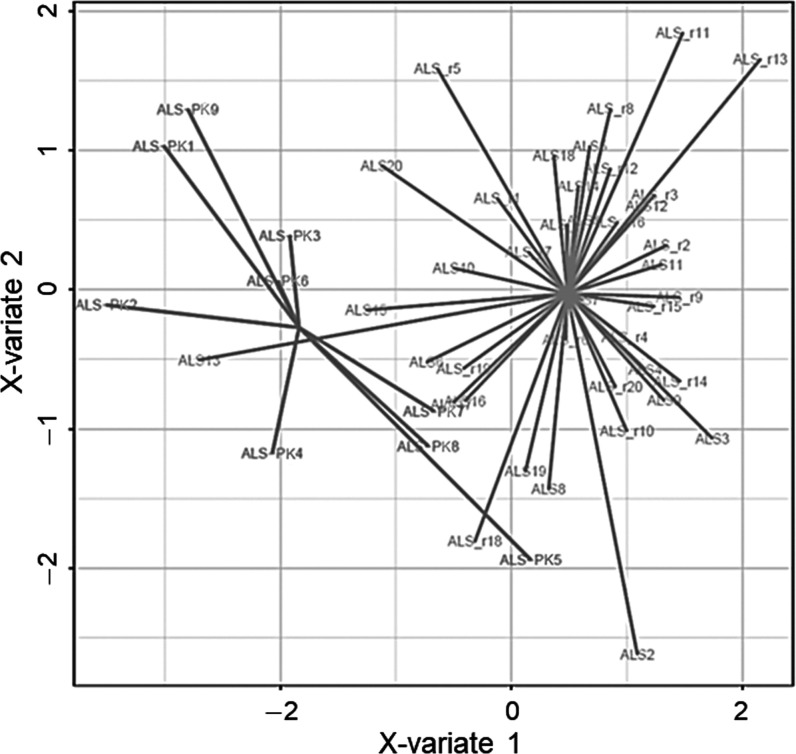
Score plot of the sPLS-DA classification based on the six-spot signature after leave-one-out cross-validation.

The residual feature set (*n* = 6) generated a discriminant model whose score plot is shown in Figure 6.

**Figure 7:**
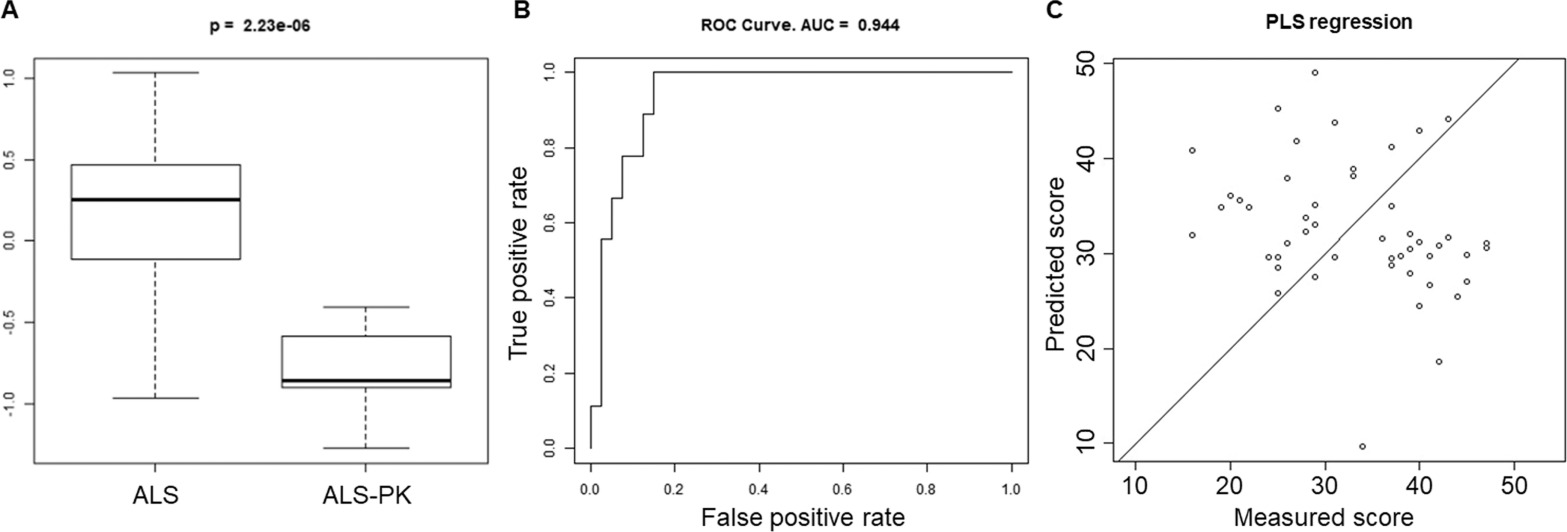
Performance of the Monte Carlo simulation model in ALS vs ALS-PK classification. Score (A) and ROC curve (B) for the best randomly generated 20-spots model. (C) Scatter plot of PLS regression showing predicted scores (three components) vs measured scores (ALSFRS-R score).

Since the above procedure failed in the identification of a consistent number of discriminating features, we decided to sample the 40 patients of the ALS group into four subsets. Each subset was used to build a sPLS-DA classifier against the 9-subjects ALS-PK group. This procedure was iterated 1000 times in a Monte Carlo simulation, and the model with the lowest *p* value was selected (Figure 7A). Subject classification using this approach showed an AUC = 0.944 (Figure 7B). Eventually, the final 20-spot protein signature was able to cluster ALS-PK patients. In order to inspect a possible correlation of the 20-spot signature with the ALSFRS-R score, a PLS regression was performed. Figure 7C shows a scatter plot of predicted vs measured scores. No correlation was observed (χ^2^ = 97; df = 48; *p* > 0.99), indicating that the severity stage of ALS cannot be inferred from the present signature. This result was expected and further supports the fact that the identified 20-spot signature includes features that are unique to the ALS-PK phenotype when compared with the main ALS group, independently of the severity of the disease.

**Figure 8:**
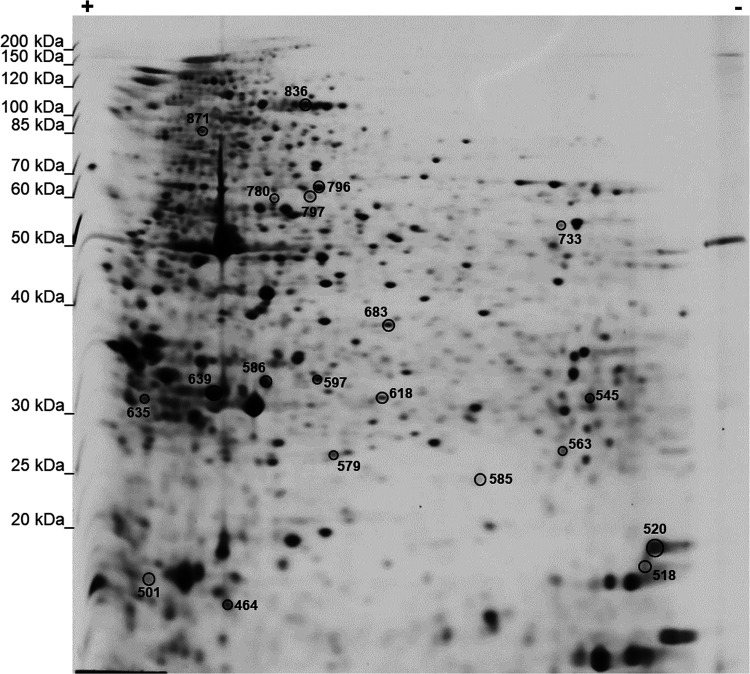
Representative 2-DE map showing the number of identifiers of the 20 spots in the signature.

Therefore, proteins corresponding to the 20 spots of the signature (Figure 8) were identified by LC-MS/MS. Table 3 reports protein identifiers for each spot as labeled in Figure 8.

**Table 3:** Identification of Protein Spots

spot number	gene	UniProt ID	protein	MW (kDa)	isoelectric point	loadings
464	COTL1	Q14019	coactosin-like protein	15.9	5.5	–0.188
501	TPM4	P67936	tropomyosin α-4 chain	28.5	4.7	0.183
518	GAPDH	P04406	glyceraldehyde-3-phosphate dehydrogenase	36.1	8.6	–0.205
520	CFL1	P23528	cofilin-1	18.5	8.2	–0.223
545	IGKC	P01834	immunoglobulin kappa constant	11.8	6.1	–0.155
563	ILK	Q13418	integrin-linked protein kinase	51.4	8.3	–0.198
579	RAB11A	P62491	ras-related protein rab-11A	24.4	6.1	–0.176
585	ENO1	P06733	α-enolase	47.2	7.0	–0.216
586	PHB1	P35232	prohibitin 1	29.8	5.6	–0.164
597	PSME1	Q06323	proteasome activator complex subunit 1	28.7	5.8	–0.207
618	ECHS1	P30084	enoyl-CoA hydratase, mitochondrial	31.4	8.3	–0.183
635	YWHAZ	P63104	14–3–3 protein zeta/delta	27.7	4.7	–0.151
639	ARPC2	O15144	actin-related protein 2/3 complex subunit 2	34.3	6.8	–0.196
683	WDR1	O75083	WD repeat-containing protein 1	66.2	6.2	–0.162
733	ENO1	P06733	α-enolase	47.2	7.0	–0.278
780	S100A7	P31151	protein S100-A7	11.5	6.3	0.273
796	PDIA3	P30101	protein disulfide-isomerase A3	56.8	6.0	–0.153
797	ALDH9A1	P49189	4-trimethylaminobutyraldehyde dehydrogenase	53.8	5.7	–0.141
836	FLNA	P21333	filamin-A	280.7	5.7	0.139
871	ACTN1	P12814	α-actinin-1	103.1	5.3	–0.166

Over-representation analysis was performed using the 20 IDs reported in Table 3. After redundancy reduction, the significantly enriched pathways resulted to be “cell–extracellular matrix interactions” and “platelet activation, signaling, and aggregation” (Table 4).

**Table 4:** Functional Enrichment Analysis Results: 20-Spot Signature (ALS vs ALS-PK)

gene set	description	size	expect	ratio	FDR
R-HSA-446353	cell–extracellular matrix interactions	18	0.031	97.71	0.0057
R-HSA-76002	Platelet activation, signaling, and aggregation	262	0.45	11.19	0.028

Compared to the previous 30-spot signature, which highlighted discriminating features between PD and ALS, this 20-spot signature highlights molecular pathways related to the presence of parkinsonian signs in ALS patients. Identified proteins and pathways mainly point to cell-to-matrix interactions and intracellular signaling as discriminating protein features of ALS-PK PBMCs compared to “classical” ALS. Therefore, also at the periphery, the biochemical processes affected in this subset of ALS patients may be different and characteristic.

Collectively, our results further support the use of PBMCs not only as a source of biomarkers but also to investigate pathobiological alterations occurring in NDs. Of interest is the possibility to discriminate between movement disorders by highlighting disease-specific pathological pathways. For instance, fibrinogen intake in PBMCs, which was originally proposed in a panel of PD peripheral biomarkers, still maintains its discriminating power also when PD subjects are compared to ALS subjects. Moreover, we demonstrated that using PBMCs, proteomics, and a global multivariate data analysis approach represents a powerful strategy to identify specific protein signatures for clinical subtypes (i.e., ALS patients with PK signs).

## 3 Methods

### 3.1 Patients Enrolment

Sixty-nine subjects were enrolled by the Parkinson’s Disease and Amyotrophic Lateral Sclerosis Centers at the Department of Neuroscience of the University of Torino, and by the Neurology Division at the Department of Translational Medicine of the University of Eastern Piedmont. One alphanumeric code was associated with each subject, to ensure that the identity was not disclosed to investigators. All patients signed informed consent before being recruited, following approval by the Institutional Review Boards of the University Hospitals where subjects were enrolled.

Patients were enrolled in two main groups: 20 subjects were *de novo* PD patients (termed here PD), and 49 were ALS patients. PD patients were diagnosed according to the Movement Disorder Society clinical diagnostic criteria for Parkinson’s Disease,^37^ and disease severity was assessed by the MDS-UPDRS. ALS functional rating scale was used for ALS patients (Table 5). Within the group of ALS patients, 20 subjects were *de novo* ALS patients (ALS_n), 20 were ALS patients already under riluzole treatment (ALS_r), and 9 were ALS patients with PK signs (ALS-PK), usually under riluzole treatment. The presence of any kind of inflammatory/infectious disease states in the 15 days before blood sampling and/or the assumption of drugs affecting peripheral blood mononuclear cells (PBMCs) were considered as exclusion criteria.

**Table 5:** Diagnostic Criteria for PD and ALS Patients

PD	ALS_n	ALS_r	ALS-PK
clinical examination	clinical examination		
MDS–UPDRS	ALSFRS-R		
brain MRI	DNA analysis for known ALS-associated gene pathogenetic variants (*SOD1*, *FUS*, *TARDBP*, *C9ORF72*)		
ioflupane I-123 SPECT.		riluzole dosage	

Information about age, sex, height, weight, smoke or alcohol consumption, work and physical activity, diagnosis, familiarity with other neurological disorders, pharmacological treatments, exposition to dangerous environmental factors, and any other recent pathologies were also collected at the time of blood sampling.

MDS-UPDRS: Movement disorder society unified Parkinson’s disease rating scale; MRI: magnetic resonance imaging; SPECT: single photon emission computed tomography; ALSFRS-R: ALS functional rating scale-revised.

Gender and age distributions were similar in the four groups. Demographic and clinical data for all patients are outlined per single group in Table 6.

**Table 6:** Outline of Patients Demographic and Clinical Information

		PD (*n* = 20)	ALS_n (*n* = 20)	ALS_r (*n* = 20)	ALS-PK (*n* = 9)
age at the withdrawal (years ± SD)		63.3 ± 10.1	67.4 ± 10.4	64.8 ± 9.8	69.8 ± 7.7
gender (percentage of males)		45%	65%	50%	67%
medication (number of unmedicated)		20	20	0	3
riluzole dosage	50 mg/d	0	0	4	0
	50 mgx2/d	0	0	16	6
ALSFRS-R score		0	37.6 ± 7.3	30.3 ± 8.4	29.2 ± 7.5
MDS-UPDRS score		36.3 ± 10.4	0	0	0

### 3.2 Peripheral Blood Mononuclear Cells Isolation

All subjects underwent a venous blood sampling (16 mL) from the antecubital vein, between 9 and 10 a.m., after an overnight fast. Blood was collected into a CPT tube with sodium citrate (BD Bioscience) and it was centrifuged (1500*g*, 20 min, room Temperature (rT)) to separate plasma, PBMCs (layer above the gel barrier), and erythrocytes/neutrophils (layer below the gel barrier). Then, the PBMC layer was transferred into a new tube and centrifuged again (10 min at 600*g*, rT). The resulting pellet was washed twice with 10 mL PBS w/o Ca^2+^ and Mg^2+^. Each sample was split into 4 aliquots and centrifuged at 500–1000*g* for 10 min. PBMCs were flash-frozen in liquid nitrogen and stored at −80 °C.

### 3.3 Two-Dimensional Gel Electrophoresis and Image Analysis

PBMCs were lysed in 100 μL of UTC buffer (7 M urea, 2 M thiourea, 4% CHAPS) added with protease and phosphatase inhibitors cocktails (Sigma-Aldrich). After 30 min incubation at rT, lysates were sonicated and centrifuged (13 000*g* for 30 min, rT) to remove cellular debris. Supernatants were collected, and protein concentration was determined according to the Bradford method. Total proteins (500 μg) were separated by two-dimensional gel electrophoresis (2-DE). First separation was performed by isoelectric focusing onto 18 cm IPG DryStrips with a nonlinear 3–10 pH gradient (GE Healthcare) using the Ettan IPGphor II Isoelectric Focusing System. Second separation was performed by SDS-PAGE on a 12.5% polyacrylamide gel. The resulting protein maps were stained with Ruthenium(II) tris (bathophenanthroline disulfonate) tetrasodium salt (Serva). RuBPS protein gel stain is highly sensitive (limit of detection: 5–10 ng), simple, and fast, it displays a wide dynamic range (3 orders of magnitude), and it is fully compatible with mass spectrometry for protein identification. Images were acquired (12-bit, grayscale) with the GelDoc-It Imaging System (UVP) and analyzed with ImageMaster 2D Platinum (GE Healthcare). A total of about 400 spots were detected and quantified.

### 3.4 Protein Spots Quantification and Selection

The volume of each spot in a map was normalized over the sum of the volumes of those spots present in all gels. The spots present in less than 65% of gels were excluded from further analysis. Then, the biological variability of the subjects in each group was assessed by Pearson linear correlation and Q-Q plots. After a logarithmic transformation data were processed to eliminate confounding factors: gender (Wilcoxon test, *p* value <0.01), age at the withdrawal (Pearson test, *p* value <0.01), and riluzole-sensitive (Student’s *t* test, *p* value <0.01) that did not correlate with ALSFRS-R score. Spots significantly correlating with confounding factors were eliminated. After the removal of riluzole-sensitive spots, a single group of ALS patients (ALS) was created, including both ALS_n and ALS_r subjects.

FGG and FGB differential expression was assessed in “ALS plus ALS-PK” vs “PD” using a two-tailed Welch Student’s *t* test.

### 3.5 Generation of a Model for Patients’ Classification

Predictive models for the classification of PD patients with respect to ALS subjects were built by sPLS-DA of all of the spots identified, after the removal of those spots whose intensity correlated with any confounding factors. Protein spots were then refined by recursive feature elimination based on the maximization of the ROC AUC following elimination of the spot with the lowest loading. Cross-validation was performed using the leave-one-out procedure (predicting one subject excluded from the model construction).

A predictive model for the classification of ALS-PK patients with respect to ALS subjects was built, as described hereafter. The 40 patients of the ALS group were sampled into four 10-patient subsets. Each subset was used to build a sPLS-DA classifier against the 9 subjects in the ALS-PK group. The mean and standard deviation of each loading were annotated, and the whole procedure was repeated 1000 times in a Monte Carlo simulation. In each simulation, the 20 spots with a lower coefficient of variation of their loadings were selected and used to calculate a score for each subject. The *p* value of a Wilcoxon test of such scores against the two groups provided a figure of merit to evaluate the performance of the classifier. The lowest *p* value observed in the 1000 simulations identified the best-performing average model that was applied, at this point, to the 40 subjects of the ALS group and to the 9 ALS-PK subjects. To verify a possible correlation of the signature with the ALSFRS-R score, a PLS regression analysis was performed, comparing predicted scores (three components) to measured scores. The significance of the correlation was assessed by a chi-square test.

All procedures for data analysis and graphics were written in R, an open-source environment for statistical computing.

### 3.6 In-Gel Digestion, LC-MS/MS, and Protein Identification

Spots corresponding to proteins of interest were excised manually from 2-DE gels. Gel bricks were washed (25 mM NH_4_HCO_3_ in 50% CH_3_CN), dehydrated (100% CH_3_CN), and proteins were reduced (10 mM DTT in 100 mM NH_4_HCO_3_ for 45 min at 56 °C) and alkylated (55 mM IAA in 100 mM NH_4_HCO_3_ for 30 min at RT in the dark). Gel bricks were then washed (25 mM NH_4_HCO_3_ in 50% CH_3_CN), dehydrated (100% CH_3_CN), and incubated for protein digestion at 37 °C overnight with 10 ng/μL trypsin in 100 mM NH_4_CO_3_ (Modified Porcine Trypsin, mass spectrometry grade, Promega). Upon digestion, peptides were extracted from gel bricks by sequential washes with 0.1% trifluoroacetic acid (TFA)/60% CH_3_CN, collected in a fresh tube, and vacuum-dried.

The peptide mixtures were separated using an LTQ XL-Orbitrap ETD equipped with an HPLC NanoEasy-PROXEON (Thermo Fisher Scientific). Protein identification was performed by searching the National Center for Biotechnology Information nonredundant database using the Mascot search engine (http://www.matrixscience.com). Input search parameters were set as follows. Enzyme: trypsin; fixed modifications: carbamidomethyl (C); variable modifications: oxidation (M), Gln → pyro-Glu (N-term Q), pyro-carbamidomethyl (N-term C); peptide mass tolerance: ± 10 ppm; fragment mass tolerance: ± 0.6 Da; maximum number of missed cleavages: 1. UniProt release 2022_03 was used as reference human protein sequence database. Proteins identified in each spot (at least 2 significant matches) were ranked based on the emPAI score, and the protein showing the highest emPAI score and protein sequence coverage was retained as protein spot identifier. Keratins and trypsin were excluded from the protein lists.

### 3.7 Functional Enrichment Analysis and PPI Network

The over-representation analysis (ORA) was carried out using the Webgestalt portal (http://www.webgestalt.org/) and Reactome as a pathway database. The reference set was the genome protein coding. Benjamini–Hochberg correction was applied. Significance was set to FDR < 0.05

The web portal String (https://string-db.org/) was used to build PPI networks. Only interactions derived from experimental and database evidence were retained. The significant network (*p* < 0.01) was further considered to interpret and discuss proteomics results. The network was exported, visualized, and modified using Cytoscape 3.9.1 (http://www.cytoscape.org/).

## Supporting Information Available

Distribution density of the sum of the volumes of all common spots (*n* = 50) in all 2-DE gels (*n* = 69) (Figure S1); distribution of missing values (Figure S2); scatter plot of a representative pair of gels belonging to the same group, quantile-quantile plot for the residuals of the linear fit (Figure S3); seven gender-sensitive spots (Figure S4); 21 age-sensitive spots (Figure S5); 13 riluzole-sensitive spots (Figure S6); protein spots quantification (Table S1); and coefficients of linear correlations of riluzole-sensitive spots with ALSFRS-R score (Table S2) (PDF)
Interindividual variability evaluated in each group (XLSX)

## Abbreviations

2-DE: two-dimensional electrophoresis
ALS: amyotrophic lateral sclerosis
ALS_n: amyotrophic lateral sclerosis *de novo* patients
ALS_r: amyotrophic lateral sclerosis patients treated with riluzole
ALS-PK: amyotrophic lateral sclerosis patients with parkinsonian signs
AUC: area under the curve
CSF: cerebrospinal fluid
FDR: false discovery rate
FTD: frontotemporal dementia
ND: neurodegenerative disease
ORA: over-representation analysis
PBMC: peripheral blood mononuclear cell
PD: Parkinson’s disease
PK: parkinsonian
PPI: protein–protein interaction
RFE: recursive feature elimination
ROC: receiver operating characteristics
rT: room temperature
sPLS-DA: sparse partial least square discriminant analysis
