# Supplementary material for: Shared and Unique Disease Pathways in Amyotrophic Lateral Sclerosis and Parkinson’s Disease Unveiled in Peripheral Blood Mononuclear Cells
Source: ACS Chem Neurosci. 2023 Nov 8;14(23):4240–51. doi: 10.1021/acschemneuro.3c00629 (PMC10704560; doi:10.1021/acschemneuro.3c00629)
Supplement: Supplementary file 1 — cn3c00629_si_001.pdf [file cn3c00629_si_001.pdf]

Supporting Information

Shared and unique disease pathways in Amyotrophic Lateral Sclerosis and Parkinson's Disease unveiled in peripheral blood mononuclear cells

Marta Lualdi [1], Federico Casale [2], Mario Giorgio Rizzone [3], Maurizio Zibetti [3], Chiara Monti [1], Ilaria Colugnat [1], Andrea Calvo [2], Giovanni De Marco [2], Cristina Moglia [2], Giuseppe Fuda [2], Cristoforo Comi [4], Adriano Chiò [2], Leonardo Lopiano [3], Mauro Fasano [1], and Tiziana Alberio [1, *]

[1] Department of Science and High Technology and Center for Research in Neuroscience, University of Insubria, Busto Arsizio (VA), Italy
[2] Neurology 1, ALS Expert Center, 'Rita Levi Montalcini' Department of Neuroscience, University of Turin, Turin, Italy, and AOU Città della Salute e della Scienza, Torino, Italy
[3] 'Rita Levi Montalcini' Department of Neuroscience, University of Torino, Torino, Italy, and AOU Città della Salute e della Scienza, Torino, Italy
[4] Department of Translational Medicine, University of Piemonte Orientale, and Sant'Andrea Hospital, Vercelli, Italy

* Correspondence: tiziana.alberio@uninsubria.it

Table S2: Coefficients of linear correlations of riluzole-sensitive spots with ALSFRS-R score.

**Spot ID**	**Coefficients ALSFRS-R**	**Pearson correlation**
498	0.037	p = 0.814
507	-0.138	p = 0.415
567	-0.130	p = 0.380
580	0.003	p = 0.986
603	0.073	p = 0.648
624	0.212	p = 0.208
631	0.356	p = 0.016
648	0.101	p = 0.513
649	-0.075	p = 0.619
681	0.105	p = 0.476
684	-0.297	p = 0.047
728	0.207	p = 0.167
787	-0.014	p = 0.935

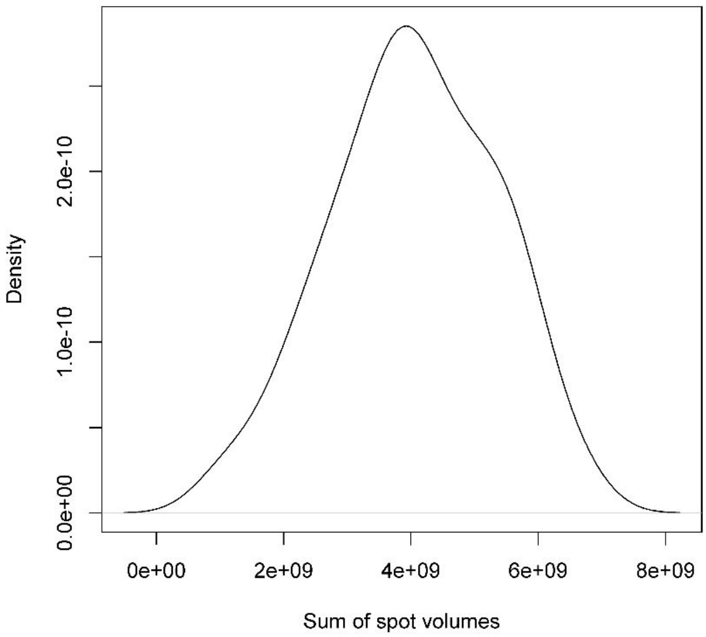

Figure S1: Distribution density of the sum of the volumes of all common spots (n=50) in all 2-DE gels (n=69).

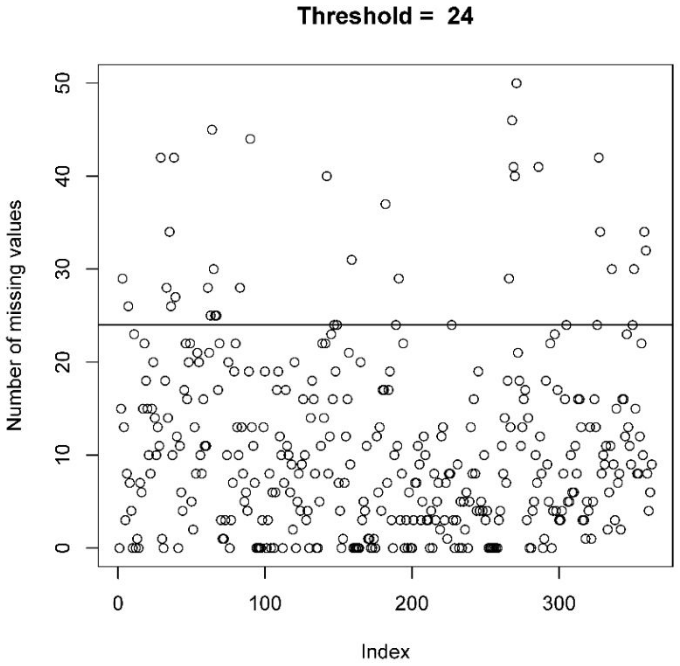

Figure S2: Distribution of missing values. Each dot represents a spot and the horizontal line represents the chosen threshold of 24 missing values. In other words, those spots that were missing in more than 24 gels out of 69 were excluded, while those that were present in at least 45 gels over 69 (65%) were retained for further analysis. Retained spots were n=324.

A B

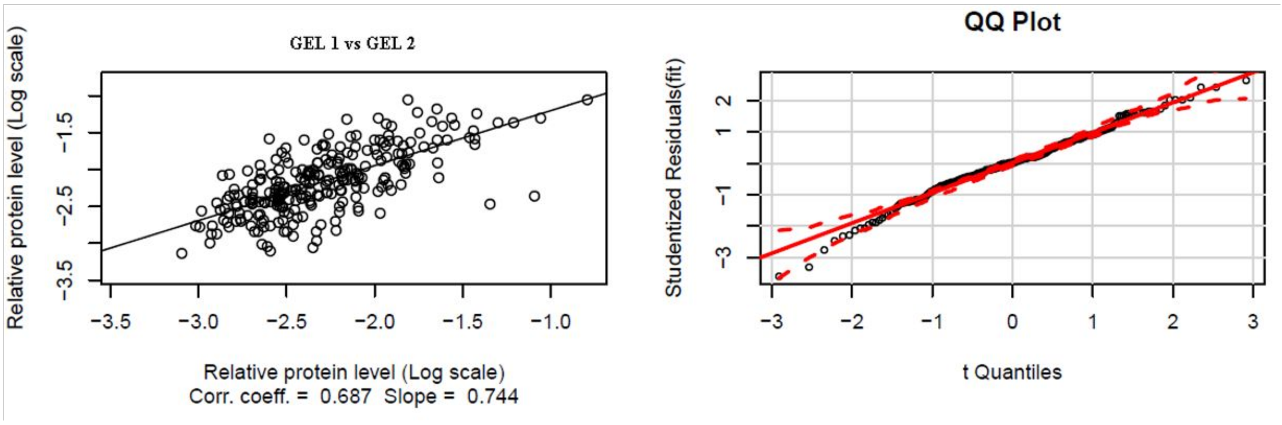

Figure S3: A) Scatter plot of a representative pair of gels belonging to the same group. The Pearson correlation coefficient is reported under the plot, together with the slope of the line. B) Quantile-quantile plot for the residuals of the linear fit. A linear correlation between *t* quantiles and studentized residuals indicates that gels are comparable.

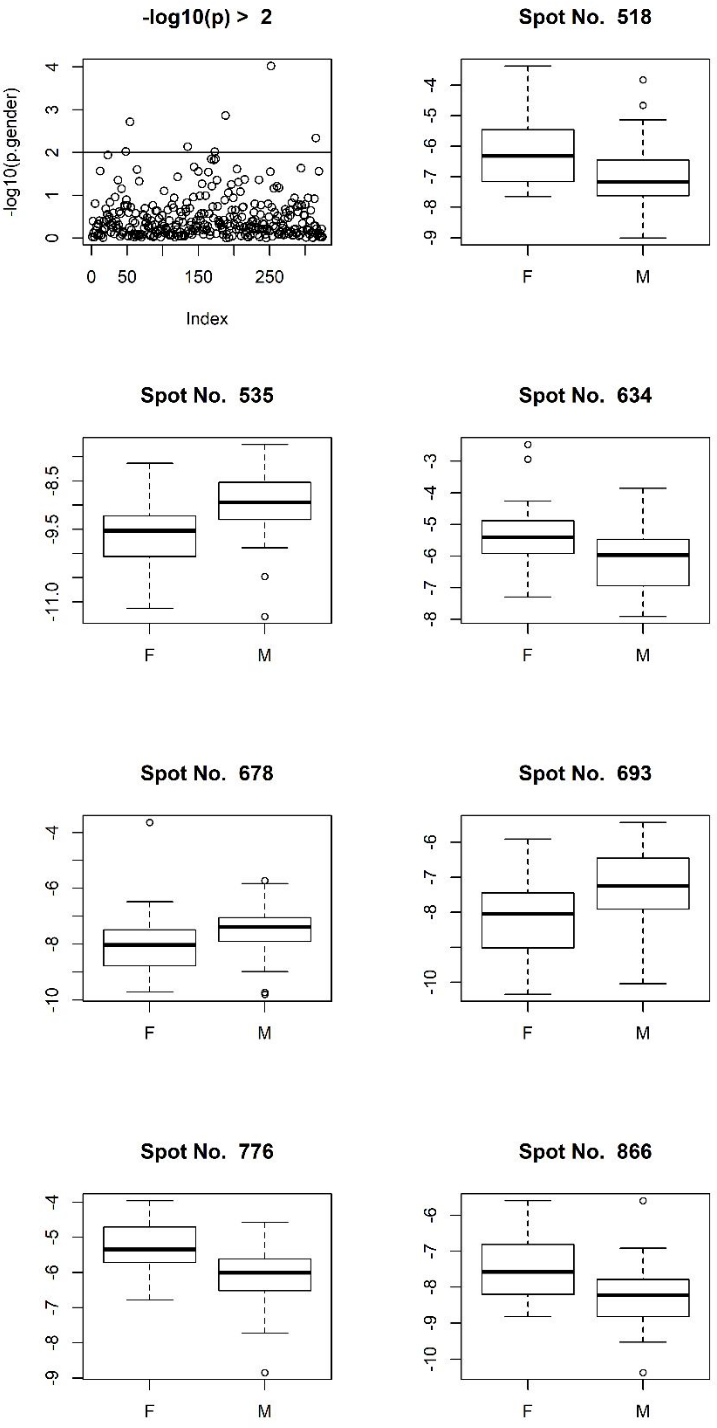

Figure S4: Seven gender-sensitive spots. Box plots represent the spots whose volumes significantly correlate with the gender by the Wilcoxon test (p<0.01). F: female. M: male.

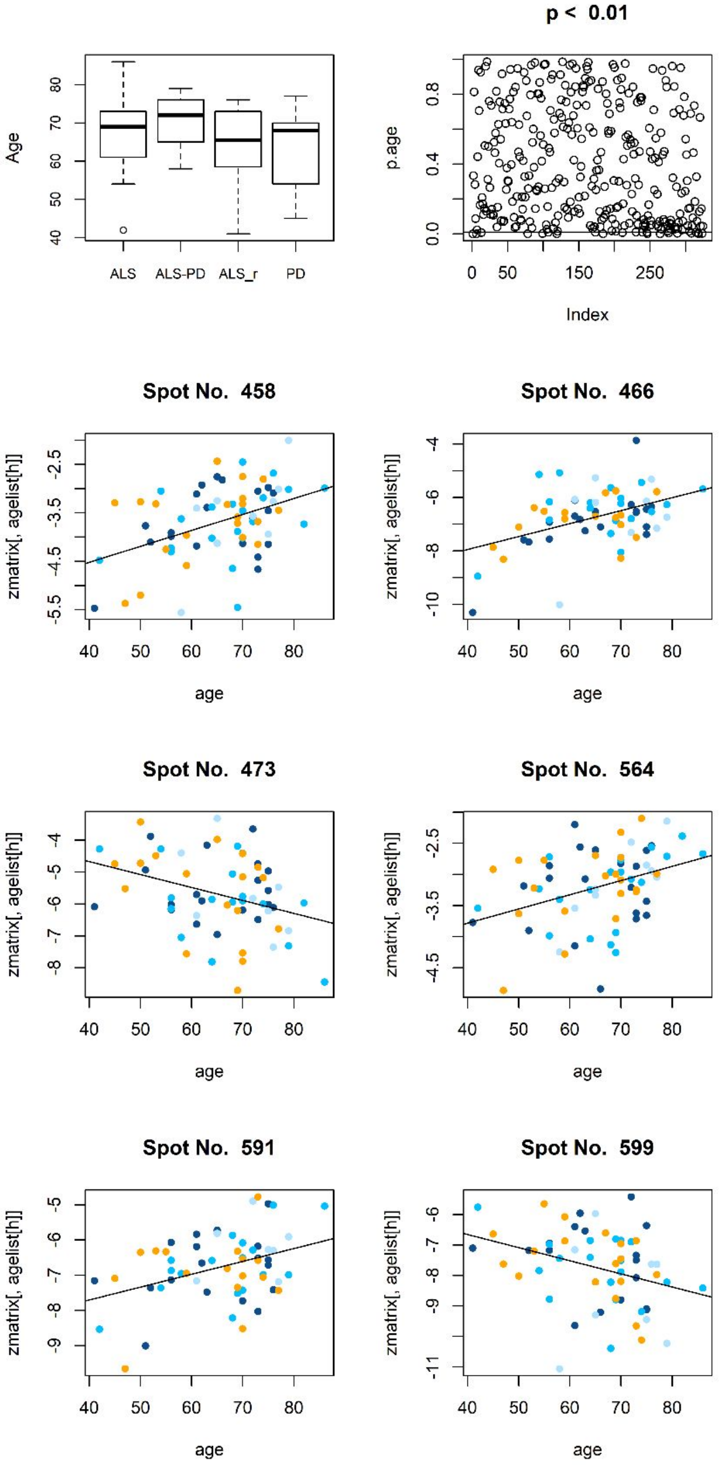

Age
ALS
ALS-PD
ALS_r
PD
p < 0.01
p.age
Index
Spot No. 458
zmatrix[, agelist[h]]
age
Spot No. 466
zmatrix[, agelist[h]]
age
Spot No. 473
zmatrix[, agelist[h]]
age
Spot No. 564
zmatrix[, agelist[h]]
age
Spot No. 591
zmatrix[, agelist[h]]
age
Spot No. 599
zmatrix[, agelist[h]]
age

continues…

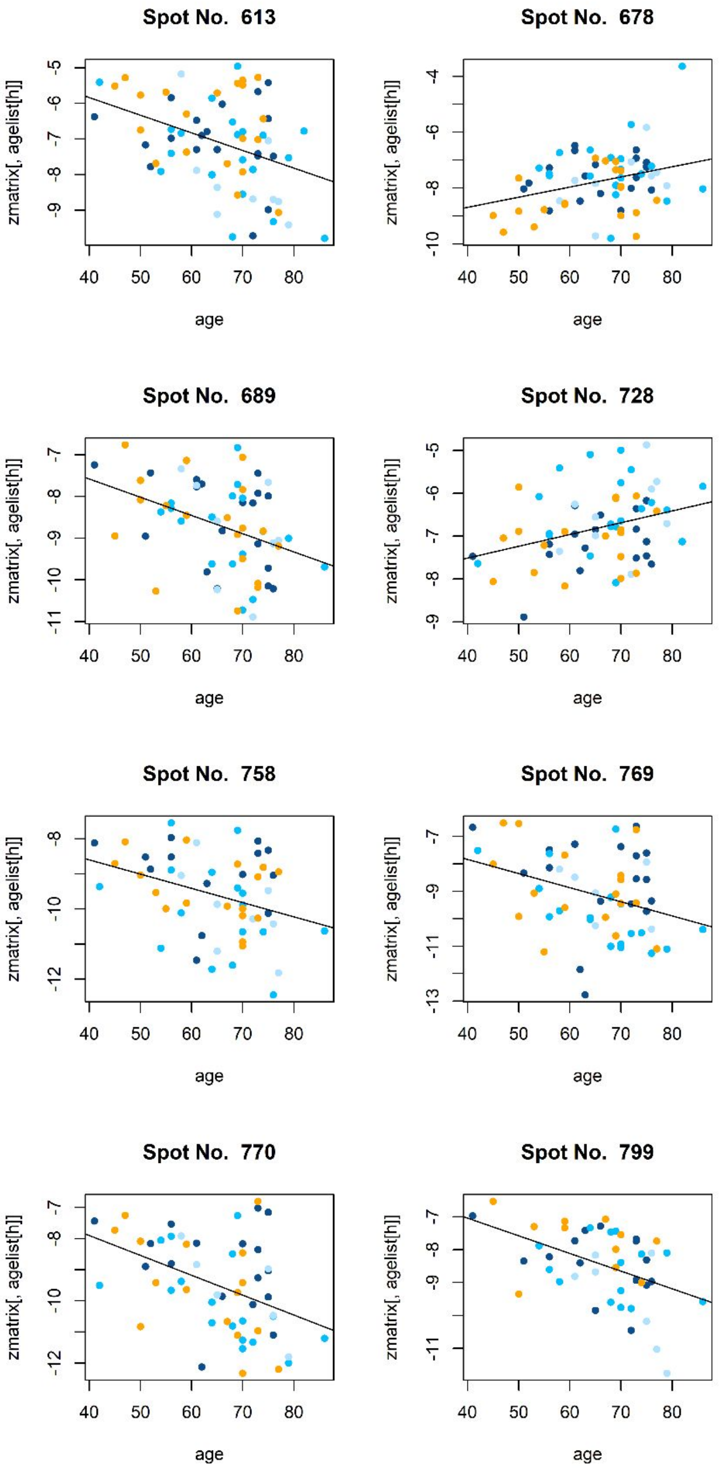
Spot No. 613
Spot No. 678
Spot No. 689
Spot No. 728
Spot No. 758
Spot No. 769
Spot No. 770
Spot No. 799
zmatrix[, agelist[h]]
age

continues…

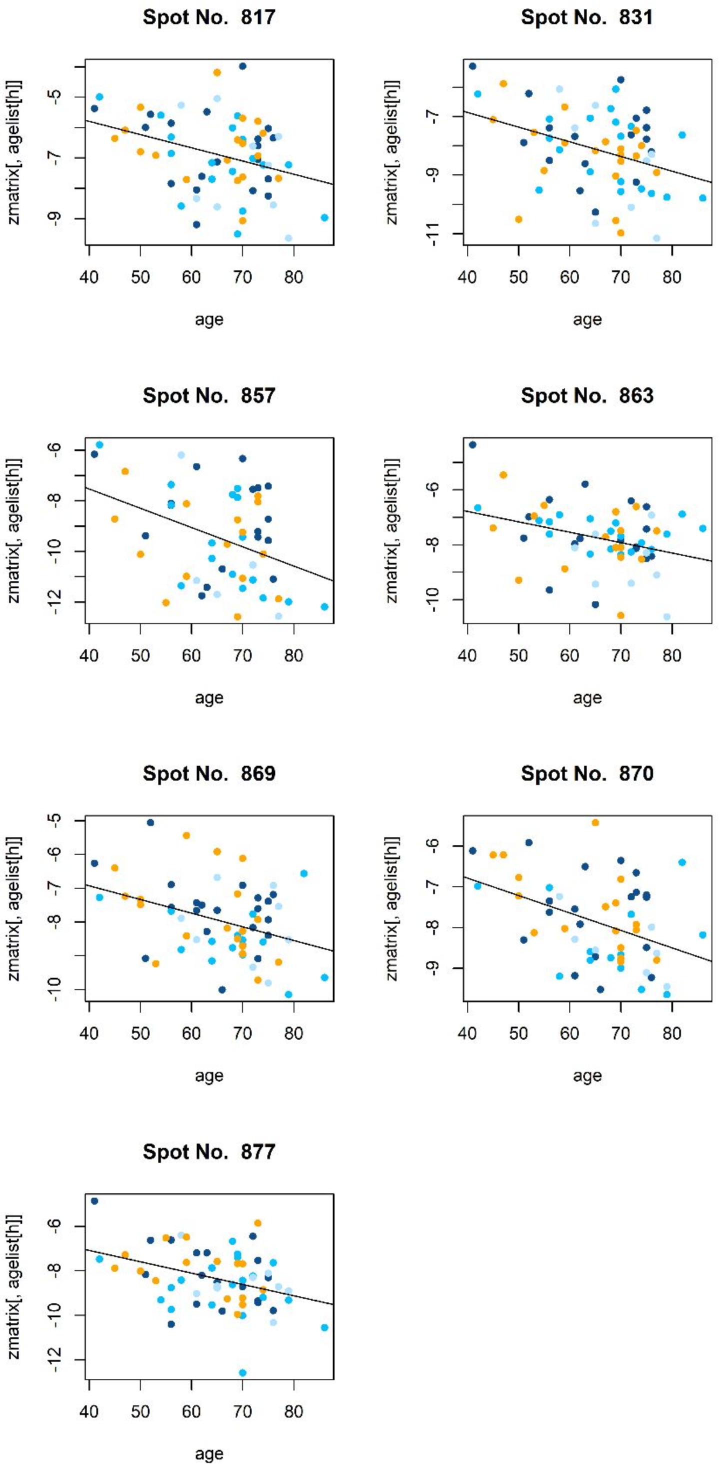

Figure S5: Twenty-one age-sensitive spots. Scatter plots represent the Pearson correlation between spot volume and age ($p<0.01$). Different colors identify the classification of subjects in different groups (Orange: PD; shades of Blue: the three ALS sub-groups).

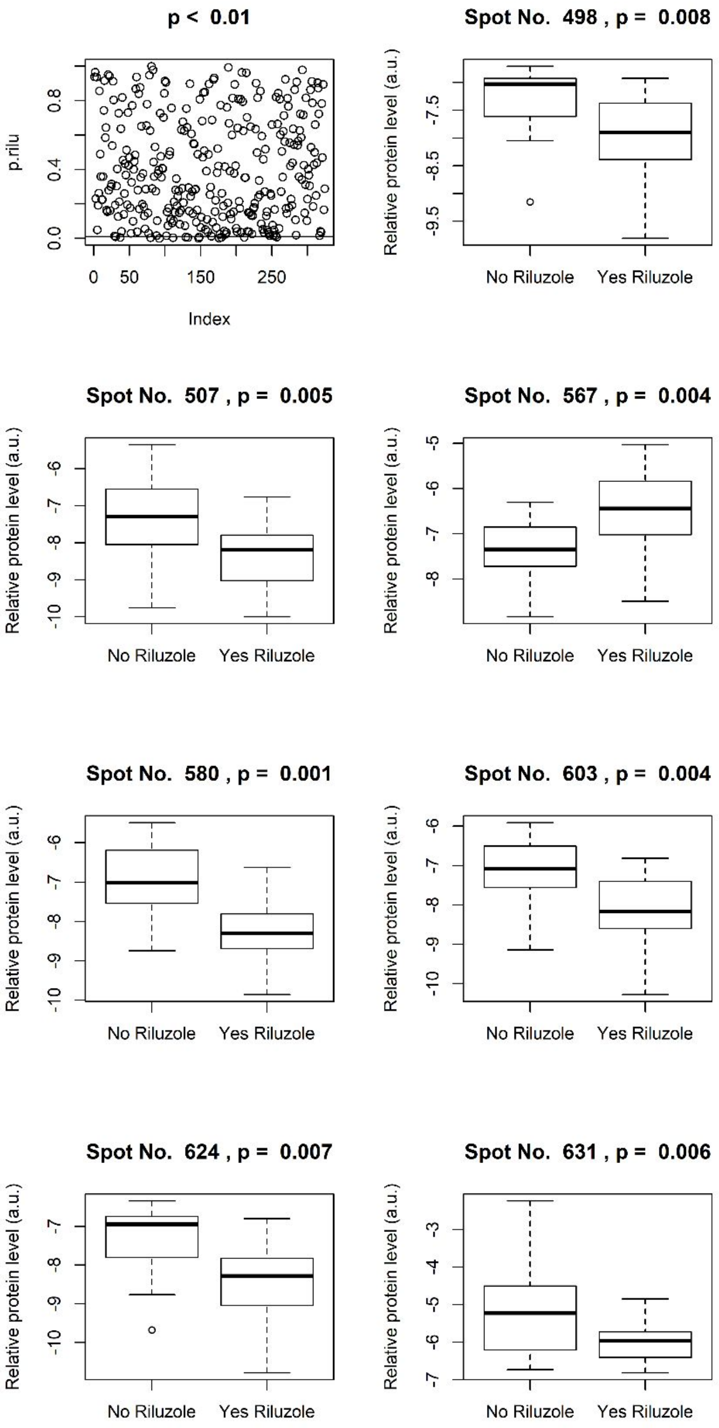
p < 0.01
p.rilu
Index
Spot No. 498 , p = 0.008
Spot No. 507 , p = 0.005
Spot No. 567 , p = 0.004
Spot No. 580 , p = 0.001
Spot No. 603 , p = 0.004
Spot No. 624 , p = 0.007
Spot No. 631 , p = 0.006
Relative protein level (a.u.)
No Riluzole
Yes Riluzole

continues…

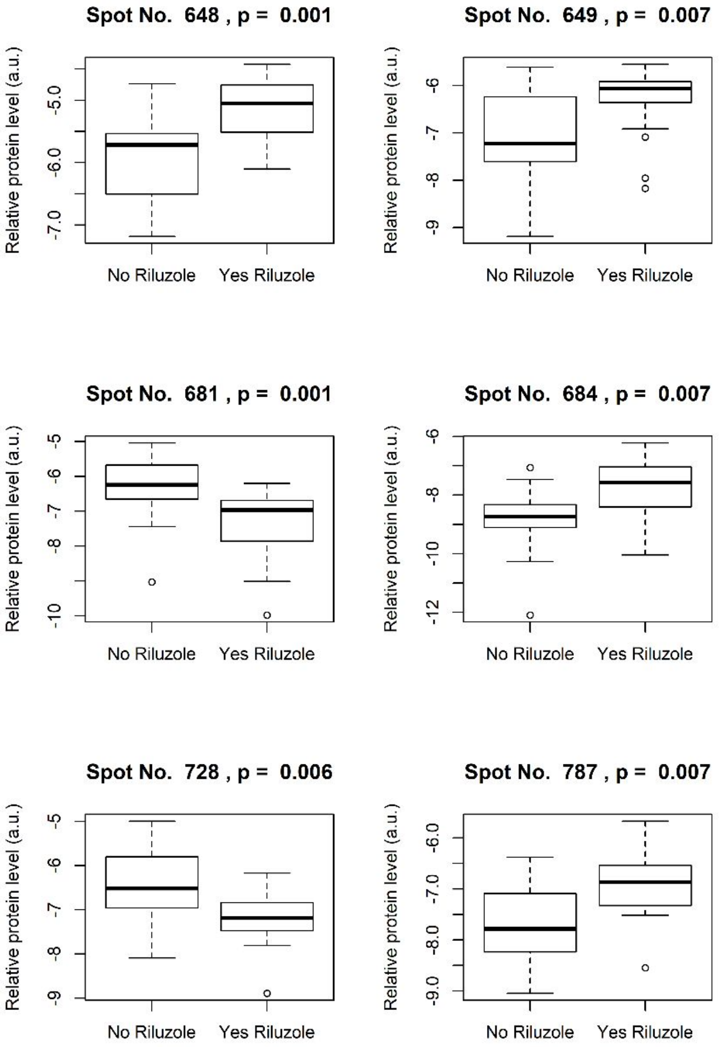

Figure S6: Thirteen riluzole-sensitive spots. Box plots represent the spots whose volumes significantly correlate with riluzole treatment by Student's *t* test ($p<0.01$).
